# Presence of Adult Companion Goats Favors the Rumen Microbial and Functional Development in Artificially Reared Kids

**DOI:** 10.3389/fvets.2021.706592

**Published:** 2021-09-07

**Authors:** Juan Manuel Palma-Hidalgo, David R. Yáñez-Ruiz, Elisabeth Jiménez, A. Ignacio Martín-García, Alejandro Belanche

**Affiliations:** ^1^Estación Experimental del Zaidín (CSIC), Granada, Spain; ^2^Department of Animal Production and Food Sciences, AgriFood Institute of Aragon (IA2), University of Zaragoza-CITA, Zaragoza, Spain

**Keywords:** bacteria, methanogens, protozoa, rumen colonization, weaning

## Abstract

Newborn dairy ruminants are usually separated from their dams after birth and fed on milk replacer. This lack of contact with adult animals may hinder the rumen microbiological and physiological development. This study evaluates the effects of rearing newborn goat kids in contact with adult companions on the rumen development. Thirty-two newborn goat kids were randomly allocated to two experimental groups which were reared either in the absence (CTL) or in the presence of non-lactating adult goats (CMP) and weaned at 7 weeks of age. Blood and rumen samples were taken at 5, 7, and 9 weeks of age to evaluate blood metabolites and rumen microbial fermentation. Next-generation sequencing was carried out on rumen samples collected at 7 weeks of age. Results showed that CTL kids lacked rumen protozoa, whereas CMP kids had an abundant and complex protozoal community as well as higher methanogen abundance which positively correlated with the body weight and blood β-hydroxybutyrate as indicators of the physiological development. CMP kids also had a more diverse bacterial community (+132 ASVs) and a different structure of the bacterial and methanogen communities than CTL kids. The core rumen bacterial community in CMP animals had 53 more ASVs than that of CTL animals. Furthermore, the number of ASVs shared with the adult companions was over 4-fold higher in CMP kids than in CTL kids. Greater levels of early rumen colonizers Proteobacteria and Spirochaetes were found in CTL kids, while CMP kids had higher levels of Bacteroidetes and other less abundant taxa (Veillonellaceae, Cyanobacteria, and Selenomonas). These findings suggest that the presence of adult companions facilitated the rumen microbial development prior to weaning. This accelerated microbial development had no effect on the animal growth, but CMP animals presented higher rumen pH and butyrate (+45%) and ammonia concentrations than CTL kids, suggesting higher fibrolytic and proteolytic activities. CMP kids also had higher blood β-hydroxybutyrate (+79%) and lower blood glucose concentrations (-23%) at weaning, indicating an earlier metabolic development which could favor the transition from pre-ruminant to ruminant after the weaning process. Further research is needed to determine the effects of this intervention in more challenging farm conditions.

## Introduction

Weaning of ruminants in natural conditions is a progressive process that occurs between 6 and 9 months of age, and it is characterized by a decrease in the frequency of suckling, with an increase in the frequency and amount of solid feed intake and development of more complex social interactions (1). However, in the current dairy production systems ruminants are weaned much earlier and newborns are typically separated from their dams immediately or during the first hours after birth, and they have no contact with adult ruminants until they are weaned or later. This practice has been shown to be stressful for both the newborn and the dam (2).

Artificial milk represents a substantial feeding cost in the current production systems, but weaning is not recommended until the rumen has a sufficient anatomical, physiological, and microbiological development (3), particularly when early weaning (conventional) is performed at 2 months of age. Several nutritional strategies have been proposed to maximize the solid feed intake prior to weaning such as decreasing milk allowance (step-down weaning) or optimizing feeder location and type of solid feed (1, 4). Nevertheless, these strategies are unlikely to favor the rumen microbial development.

Microbiota present in several maternal-associated sources, such as colostrum, vagina, udder skin, and saliva, has been reported to be able to colonize the gastrointestinal tract (GIT) of newborn ruminants within the first days of life (5). However, ruminants are born protozoa-free (afaunated) and rumen protozoa only get established after a direct and continuous nose–nose contact with adult animals (6). Therefore, under the isolation farm conditions described above, an optimal microbial GIT colonization in the newborns may be jeopardized (7, 8), having negative consequences on the transition from milk to solid feed diet throughout the weaning process (9, 10). The use of pre- and probiotics has been gaining importance in the livestock sector in the twenty first century (11) to improve animal performance and to prevent the growth of pathogenic microorganisms, promoting a more favorable microbial community in the GIT (12). Previous works have evaluated the effects of providing individual or a simple mix of microorganisms, specially *Saccharomyces cerevisiae* yeast, that are not autochthonous from the rumen or the GIT altogether (13, 14). As a result, in recent years, several authors have explored the inoculation of GIT autochthonous microbial strains (e.g., *Lactobacillus, Enterococcus, Megasphaera*, etc.) and rumen microbiota from adult animals to young ruminants (9, 15–18). A repetitive oral inoculation of a mature rumen microbiota has shown to accelerate the rumen microbial colonization and increase microbial diversity in the rumen of goat kids (10). This strategy resulted in a less severe weaning process in terms of average daily gain (ADG) mainly because inoculated animals started consuming solid feed earlier than those without inoculation (9). However, this microbial inoculation may not be feasible in commercial farms due to veterinary regulations, animals' health concerns and well-being issues, and/or the inherent difficulty to obtain fresh rumen fluid to be used as inoculum to maximize its efficacy (19). A potential approach to circumvent this limitation is to house young animals with older weaned companions that would serve as main sources of microbiota. De Paula Vieira et al. (20) demonstrated that the presence of adult companions stimulated the feeding social learning of feeding and reported improved concentrate feed intake and growth rate before and after weaning in calves. However, the impact of such intervention on the rumen function and microbial development using next-generation sequencing has not been investigated under modern dairy management practices.

The present study builds upon this existing knowledge and aims to further explore whether the presence of non-lactating adult goats (as companions) could act as natural source of microbes favoring the rumen microbial colonization in young ruminants. We hypothesized that the sole contact between adult and young animals would allow a microbial transfer, leading to an acceleration in the rumen microbial development and its function with potential positive effects during the weaning process to be further implemented under on farm conditions.

## Materials and Methods

### Experimental Design

Animal procedures conducted in this work were approved by the Ethical Committee for Animal Research (EEZ-CSIC) and carried out by trained personnel according to the Spanish guidelines (RD 53/2013). A total of 32 Murciano-Granadina newborn goats were used in this experiment. After parturition, goat kids were separated from their dams, weighed, and fed with ~250 ml of natural colostrum in two separate doses as previously described (9). Goat kids were randomly allocated to two experimental groups (16 animals each), keeping a similar average body weight (BW) and male/female ratio in both groups. One group was used as control (CTL) and kept isolated from any contact with adult animals throughout the whole experiment (conventional practice), while the other group was in continuous contact with two non-lactating goats as companion (CMP). These adult goats belonged to a healthy experimental herd and were dewormed (Ivomec^®^ Oral, Boehringer Ingelheim, Barcelona, Spain) 1 week before the beginning of the trial. Both groups had free access to water (CMP kids shared water through with the adults) and a milk replacer at 170 g/ml (Univet^®^ Spray, NutralSCA, Colmenar Viejo, Spain). From day 14, all animals had *ad libitum* access to starter concentrate (0–18 Granulado Arranque Pequeños Rumiantes Pulmorex, Macob, Granada, Spain) and oat hay until week 9. The chemical composition of these feeds is reported in Supplementary Table 1. The amount of starter concentrate offered to each group was recorded throughout the experiment. In the CMP group, a fence only traversable by goat kids was placed to prevent adult goats to have access to milk replacer and starter concentrate. Adult goats were fed with oat hay *ad libitum* and a limited amount of the starter compound feed (300 g DM/day). Milk feeding of the kids was stopped abruptly at 7 weeks of age. Body weight was monitored weekly from birth until the end of the experiment at week 9.

### Sampling and Analyses

Blood and rumen samples were collected before the morning feeding (09:00 h) at 5, 7, and 9 weeks of age, representing the pre-weaning, weaning, and post-weaning stages, respectively. Blood samples were centrifuged at 2,000 × g for 15 min, and supernatant was stored at −80°C for β-hydroxybutyrate (BHB), glucose, urea, and total protein determination as previously reported (9). Rumen content from each animal was collected by orogastric intubation (~30 ml) as previously described (21). Rumen content was filtrated through a double layer of cheesecloth to discard solid debris, and pH was immediately recorded. Similarly to how it is described in Belanche et al. (9), three subsamples of 0.8 ml were taken: the first sample was diluted with 0.2 ml of trichloroacetate solution (25 g/l) for ammonia determination. The second sample was diluted with 0.8 ml of an acid solution (0.5 mol/l HCl, 200 g/l metaphosphoric acid and 0.8 g/l crotonic acid as internal standard) for volatile fatty acid (VFA) determination. The third sample was diluted with 0.8 ml of formaldehyde solution (8% v/v) for protozoal counting. The ammonia concentration was determined by a colorimetric method (22). Individual and total VFA were measured using a gas chromatograph with a flame ionization detector (Auto-System, Perkin Elmer, Waltham, MA, USA), and protozoal counting and classification were visually determined in 15 μl of rumen liquid (23) using an optical microscope (Nikon Labophot, Tokyo, Japan). An additional subsample of rumen fluid was snap-frozen in liquid N and stored at −80°C for DNA extraction. These subsamples were then freeze-dried and bead-beat for 1 min (Mini-BeadBeater, BioSpec Products, Bartlesville, OK, USA), and DNA was extracted using a commercial kit (QIAamp DNA Stool Mini Kit, Qiagen Ltd., Barcelona, Spain). Rumen content from the two adult companions was also sampled by orogastric intubation at the beginning of the experiment for protozoal counting and DNA extraction using the same procedures. Negative controls for the DNA extraction and sequencing were also included.

Eluted DNA (2 μl) was used to assess the abundance of the main microbial groups by quantitative PCR (qPCR) using an iQ5 multicolor Real-Time PCR Detection System (Bio-Rad Laboratories Inc., Hercules, CA, USA) as described by Abecia et al. (24). Specific primers for the 16S bacterial rRNA gene, mcrA gene for methanogenic archaea, and 18S rRNA genes for protozoa and anaerobic fungi were used as previously described and validated [(25–28); respectively]. Cycling conditions were 95°C for 5 min; 40 cycles of 95°C for 15 s, 60°C for 30 s, and 72°C for 55 s; and 72°C for 1 min. The absolute amount of each microbial group, expressed as the corresponding gene copies/g of dry matter, was determined using serial dilutions of standards. The standards consisted of the plasmid PCR 4-TOPO (Invitrogen, Carlsbad, CA, USA), with an inserted 16S, mcrA, or 18S rRNA gene fragment from each microbial group, respectively.

### Next-Generation Sequencing

The structure of the rumen prokaryotic community was explored at weaning (week 7) using a meta-taxonomic approach. Eight kids from each treatment were randomly selected, and a template of the extracted DNA was sent to the Genomics Service at Instituto de Parasitología y Biomedicina López Neyra (IPBLN-CSIC, Granada, Spain) for amplicon sequencing using the MiSeq V3 (600 cycles) kit (Illumina Inc., San Diego, CA, USA). The prokaryotic universal primers used for the amplification were Pro341F 5′-CCTACGGGAGGCAGCAG-3′ and Pro805R 5′-GACTACNVGGGTATCTAATCC-3′ targeting the V3_V4 hypervariable region of the 16S rRNA gene including bacteria and methanogenic archaea (29). Primer-sorted and demultiplexed paired-end reads were used separately for bacteria and methanogens, and downstream processing was performed using QIIME 2 (30). Low-quality reads and bases (PHRED quality score below 25) were trimmed. Chimeras were identified and removed using chimera.vsearch. Amplicon sequence variants (ASVs) were identified, and then representative sequences from all ASVs were aligned against Greengenes 13_8 97% for bacteria (31) and RIM-DB for archaea (32). Once alignment was performed, data from each of the two major microbial groups were processed separately. The number of sequences per sample for each microbial group was normalized across all the samples, and singletons were removed. Raw sequence reads were deposited at the European Nucleotide Archive repository (accession: ERP126589) (33).

### Statistical Analyses and Calculations

Statistical analyses were conducted using SPSS software (IBM Corp., Version 26.0, New York, NY, USA). Rumen fermentation parameters, blood metabolites, quantitative PCR, and BW data were analyzed based on a repeated-measure mixed-effect ANOVA as follows:

$$\begin{array}{l} Y_{ijklm}=\mu +C_{i}+T_{j}+CT_{ij}+A_{k}+e_{ijkl} \end{array}$$

where *Y*_*ijkm*_ is the dependent, continuous variable, μ is the overall population of the mean, C_*i*_ is the fixed effect of the presence of companion goats (*i* = CTL vs. CMP), *T*_*j*_ is the fixed effect of the time (*j* = 5 vs. 7 vs. 9 weeks of age), *CT*_*ij*_ is the interaction term, *A*_*k*_ is the random effect of the goat kids (*k* = 1 to 32), and *e*_*ijkl*_ is the residual error. When significant effects were detected, means were compared by Fisher's protected LSD test. Quantitative PCR data and protozoal optical count data were log10 transformed before the analysis to achieve a normal distribution. Bacterial and methanogen diversity indexes at week 7 were analyzed using an ANOVA test with the treatment (CTL vs. CMP) as fixed effect. Microbial data were analyzed following the overall approach described by Belanche et al. (34). Microbial taxa abundances were analyzed with the Kruskal–Wallis non-parametric test, given that data did not have a normal distribution after performing the Shapiro–Wilk test. Only taxa with relative abundance > 0.05% were shown. In all analyses, significant effects were declared at *p* < 0.05, tendency to difference at *p* < 0.1.

Venn diagrams were performed to illustrate the treatment effects on the core microbial community, defined as the number of ASVs shared across the majority (>75%) of the individuals within each group. Venn diagrams also included the microbial community found in the two adult companion goats which were considered as a potential source of rumen microbiota. To illustrate the treatment impact on the rumen prokaryotic community, a permutation-based analysis of variance (PERMANOVA) including *p-*values and similarity was calculated based on the Bray–Curtis distance matrix. To achieve this, the log10 transformed bacteria and methanogen sequencing data were submitted to 999 random permutations under the reduced model and the Monte Carlo method (34) using PRIMER-6 software (PRIMER-E Ltd., Plymouth, UK). Pair-wise comparisons were performed to compare the microbial composition across treatments. Principal coordinate analyses (PCoA) were carried out to show the effects on the bacterial and methanogen rumen community structure. Tripod vectors were included in the PCoA to identify the most discriminant ASVs (based on Spearman correlation > 0.8). Additional Spearman correlations (ρ) were calculated to assess the relationships between the microbial taxa abundance (log10 number of sequences) and the rumen fermentation and blood parameters. Strong correlations were defined as those with ρ ≥ 0.4 or ≤ −0.4 and *p* < 0.01.

## Results

### Animals' Growth, Rumen Fermentation, and Blood Metabolites

The presence of adult companions had no effect on the animal performance in terms of BW and ADG from birth to 9 weeks of age (Table 1). Similarly, both experimental groups had similar starter DMI until week 9 (average 6.25 kg per goat kid). The rumen fermentation pattern was highly affected by the age of the kids leading to a progressive increase in VFA concentration and propionate and butyrate molar proportions, whereas rumen ammonia and acetate molar proportions decreased over time (*p* < 0.01). CMP animals also had higher rumen pH (*p* = 0.046) and branched-chain VFA (i.e., iso-valerate and iso-butyrate, *p* = 0.033) than CTL kids. The butyrate molar proportion showed an interaction (*p* < 0.001) since the higher values observed in CMP than in CTL kids increased with age, leading to substantial increments at 7 (+23%) and 9 weeks of age (+83%). The concentrations of all measured blood metabolites presented significant differences according to the sampling time (Table 1). The concentration of BHB and urea increased (*p* < 0.05) while glucose concentration decreased over time (*p* = 0.013). The blood BHB concentration in CMP goat kids was higher than in CTL across all sampling times (*p* = 0.048). The ratio BHB/glucose also increased with age (*p* = 0.013) and tended to be higher in CMP than in CTL kids (*p* = 0.088).

**Table 1:** Rumen fermentation and blood parameters of goat kids reared in the absence (CTL) or presence of adult companions (CMP) at 5, 7, and 9 weeks of age (*n* = 16).

**Time**	**Week 5**		**Week 7**		**Week 9**			***p*** **-value**		
**Treatments**	**CTL**	**CMP**	**CTL**	**CMP**	**CTL**	**CMP**	**SEM**	**Treat**.	**Time**	**T × T**
BW, kg	6.53	6.66	8.33	8.39	9.49	9.15	0.208	0.980	<0.001	0.124
Rumen pH	6.53	6.65	6.42	6.55	7.00	7.08	0.036	0.046	<0.001	0.775
NH_3_-N, mg/dl	17.1	18.1	14.8	13.7	2.74	7.93	0.883	0.279	<0.001	0.158
VFA, mM	25.0	23.9	31.8	32.4	30.6	36.4	1.160	0.452	0.002	0.193
Acetate, %	74.7	72.5	71.4	69.0	64.0	63.4	0.723	0.158	<0.001	0.576
Propionate, %	13.5	13.7	16.1	16.6	19.0	17.1	0.374	0.515	<0.001	0.229
Butyrate, %	4.83^d^	6.06^cd^	7.04^c^	8.68^b^	6.28^c^	11.5^a^	0.298	<0.001	<0.001	<0.001
Valerate, %	2.29	2.22	2.09	2.02	2.04	2.02	0.051	0.955	0.039	0.534
Isobutyrate, %	2.04	2.69	1.50	1.59	2.29	2.38	0.101	0.187	0.920	0.263
Isovalerate, %	2.61^bc^	2.91^ab^	1.92^d^	2.12^cd^	2.21^cd^	3.40^a^	0.103	0.009	0.831	0.049
BCVFA^1^, % %	4.64	5.59	3.36	3.68	4.50	5.78	0.187	0.033	0.957	0.685
**Blood metabolites**										
BHB, mM	0.97	1.49	1.34	2.40	2.69	3.18	0.198	0.048	0.015	0.935
Glucose, mg/dl	105	94.0	101	78.1	73.8	82.6	3.020	0.052	0.008	0.082
Ratio BHB/glucose	0.18	0.38	0.28	0.72	0.72	0.70	0.069	0.088	0.013	0.354
Urea, mg/dl	16.1	21.1	24.0	17.9	35.0	40.3	1.660	0.479	0.001	0.705
Total proteins, g/l	53.1	53.4	61.0	59.4	57.1	59.6	0.746	0.554	0.052	0.297

a−d *Means within a row with different superscripts differ (p < 0.05)*.

1 *Branched-chain VFA= iso-valerate + iso-butyrate*.

### Rumen Microbial Community

Quantitative PCR analysis showed that the ruminal concentrations of bacteria and fungi were unaffected by the experimental treatment (Table 2). The methanogen concentration was higher in CMP than in CTL kids at 5 weeks of age, but differences tended to become smaller over time (interaction, *p* = 0.039). The largest differences promoted by the presence of adult companions were related to the protozoal community: CTL kids were protozoa-free (afaunated) since no protozoal cells were detected with optical microscope examination, and qPCR showed negligible concentrations of protozoal DNA (1,000 folds lower than CMP kids). On the contrary, CMP kids showed an abundant (from 4.96 to 5.21 log10 cells/ml) and diverse protozoal community dominated by the subfamily *Entodiniinae* but also with other protozoal groups such as *Diplodiniinae, Ophryoscolex, Isotricha*, and *Dasytricha*. These protozoal groups were also present in both of the adult companions, *Entodiniinae* being the most abundant group (Supplementary Table 2). The rumen protozoal community in the CMP animals also developed over time, promoting an increase in the protozoal concentration, both in DNA gene copies (*p* < 0.001) and in cells (*p* = 0.012), and a substantial increase in the proportions of *Ophryoscolex* (*p* < 0.001), *Isotricha*, and *Dasytricha* in detriment of the subfamily *Entodiniinae*.

**Table 2:** Abundance of the major rumen microbial groups in goat kids reared in the absence (CTL) or presence of adult companions (CMP) at 5, 7, and 9 weeks of age (*n* = 16).

**Time**	**Week 5**		**Week 7**		**Week 9**			***p*** **-value**		
**Treatments**	**CTL**	**CMP**	**CTL**	**CMP**	**CTL**	**CMP**	**SEM**	**Treat**.	**Time**	**TxT**
**Microbes, log10 copy/g DM**										
Bacteria	9.14	9.34	9.26	9.31	9.27	9.33	0.036	0.149	0.28	0.251
Methanogens	6.18^c^	6.77^a^	6.35^bc^	6.65^ab^	6.22^c^	6.37^bc^	0.051	0.020	0.467	0.039
Protozoa	<4.0^c^	6.87^b^	<4.0^c^	7.25^ab^	<4.0^c^	7.62^a^	0.168	<0.001	<0.001	0.077
Anaerobic fungi	4.96	4.77	5.16	5.10	4.42	4.08	0.088	0.955	0.279	0.180
Protozoa, log10 cell/ml	ND	4.96	ND	5.12	ND	5.21	0.260		0.125	
Subf. *Entodiniinae*, %	ND	95.3	ND	94.2	ND	89.8	4.740		0.598	
Subf. *Diplodiniinae*, %	ND	4.37	ND	4.65	ND	6.28	0.403		0.315	
*Ophryoscolex* spp., %	ND	0.30^b^	ND	0.93^b^	ND	3.20^a^	0.161		<0.001	
*Isotricha* spp., %	ND	0^b^	ND	0.19^ab^	ND	0.54^a^	0.051		0.056	
*Dasytricha* spp., %	ND	0^b^	ND	0.05^ab^	ND	0.18^a^	0.017		0.062	

a−c *Means within a row with different superscripts differ (p < 0.05). ND, not detected*.

### Sequencing Analysis

**Figure 1:**
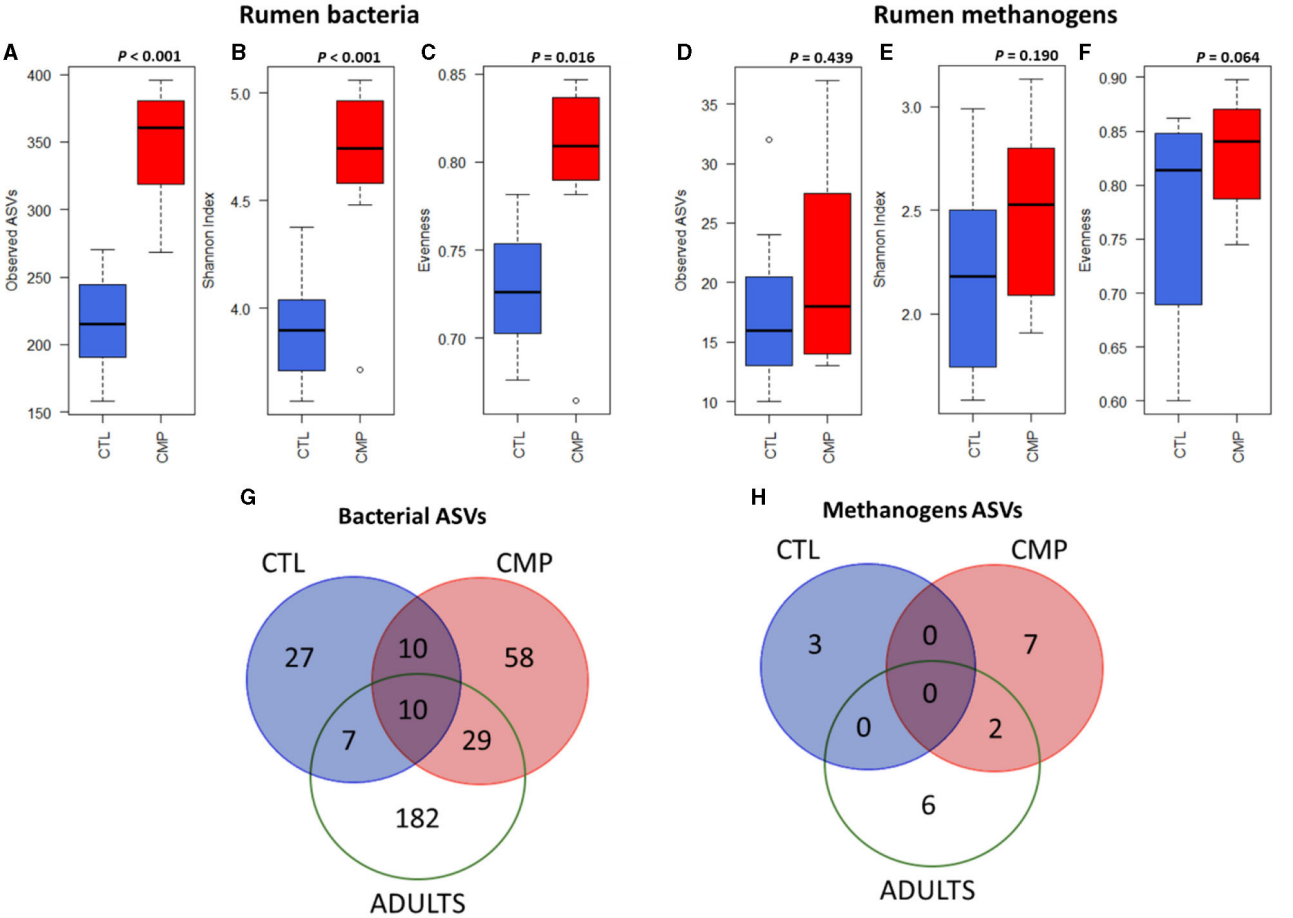
Boxplots indicating the rumen bacterial diversity in terms of observed ASVs **(A)**, Shannon index **(B)**, and evenness **(C)** and the rumen methanogen diversity (**D–F**; respectively) in goat kids (*n* = 8) reared in absence (CTL) or presence of adult companions (CMP) at 7 weeks of age. Venn diagrams of the core rumen bacterial **(G)** and methanogen **(H)** communities (present in >75% of animals in each group) at ASV level in CTL and CMP goat kids at 7 weeks of age, along with the two adult companions (Adults).

The sequencing analysis generated 30,151 ± 9,600 high-quality prokaryotic sequences per sample. The number of sequences was normalized to 20,551 for further processing and analyses. The Good's coverage for the bacterial and methanogen communities was 99 and 70%, respectively. The rumen bacterial diversity in terms of observed ASVs, Shannon index, and evenness was much higher in CMP than in CTL goat kids at weaning (*p* < 0.001, *p* < 0.001, and *p* < 0.05; respectively) (Figures 1A–C; Table 3). CMP kids also tended to have a more diverse methanogen community in terms of evenness and Simpson index (*p* < 0.1), but no differences were observed for number of ASVs or Shannon index (Figures 1D–F; Table 2).

**Table 3:** Abundance of rumen bacterial taxa in the rumen of goat kids reared in absence (CTL) or presence of adult companions (CMP) at 7 weeks of age (*n* = 8).

	**Treatments**				
	**CTL**	**CMP**	**Adults^a^**	**SEM**	***p*** **-value^b^**
*p_Actinobacteria, f_Coriobacteriaceae*	0.12	0.04	0.82	0.025	0.079
*p_Bacteroidetes*	64.2	71.5	62.6	1.960	0.093
*f_Paraprevotellaceae*	3.23	5.77	2.40	0.704	0.059
*f_Bacteroidaceae, g_BF311*	0.18	0.62	1.57	0.108	0.027
*p_Cyanobacteria*	0.02	0.08	0.05	0.164	0.021
*p_Firmicutes*	16.6	13.7	21.4	0.837	0.059
*f_Ruminococcaceae*	7.31	5.09	5.71	0.705	0.093
*f_Veillonellaceae*	0.68	1.40	1.21	0.184	0.027
*g_Selenomonas*	0.14	0.33	0.15	0.068	0.035
*g_Succinispira*	0.01	0.69	0.85	0.142	0.001
*f_Erysipelotrichaceae*	1.03	1.47	0.75	0.269	0.046
*g_Bulleidia*	0.00	0.12	0.05	0.036	0.004
*p_Proteobacteria*	4.24	2.23	0.86	1.040	0.338
*f_Rhodocyclaceae*	0.21	0.05	0.02	0.048	0.059
*g_Georgfuchsia*	0.21	0.05	0.00	0.048	0.046
*f_Desulfovibrionaceae*	0.44	0.24	0.44	0.057	0.036
*p_Spirochaetes*	6.57	2.09	0.70	1.220	0.021
*f_Spirochaetaceae*	5.05	1.25	0.41	1.020	0.021
*g_Spirochaeta*	0.02	0.55	0.13	0.012	0.062
*g_Treponema*	2.04	0.58	0.15	0.389	0.036
*p_SR1*	0.00	0.11	0.26	0.038	0.001
*p_Synergistetes*	0.07	0.19	0.18	0.034	0.059
*g_Dethiosulfovibrio*	0.01	1.20	0.72	0.035	0.004
*p_Tenericutes*	0.04	0.10	0.11	0.022	0.088
*p_TM7, f_F16*	0.08	0.23	0.04	0.037	0.021
*p_Verrucomicrobia*	1.28	3.35	0.76	0.620	0.074
*f_RFP12*	0.59	2.95	0.60	0.577	0.016

a *Description of the rumen bacterial community in the adult companions*.

b *p-values for the differences between CTL and CMP kids. Only taxa with an average abundance > 0.05% are shown. p, phylum; f, family; g, genus*.

The Venn's diagram (Figure 1G) showed that the number of ASVs that made the core rumen bacterial community in each group of animals (ASVs present in >75% animals within each group) was greater in CMP than in CTL (107 vs. 54 ASVs). Among those ASVs, only 20 of them were shared between CMP and CTL kids. The two adult goats had the largest rumen bacterial core community (228 ASVs), and although most of them were adult goats-specific, a larger proportion of them was specifically shared with CMP than with the CTL kids (29 vs. 7 ASVs). Out of the 29 ASVs shared between CMP kids and the adults, 15 belonged to the *Bacteroidales* order and 8 to *Clostridiales* order. Only 10 bacterial ASVs were shared across CTL, CMP, and adult goats. A similar core community pattern, but with much lower numbers, was found regarding the core rumen methanogens ASVs (Figure 1H).

**Figure 2:**
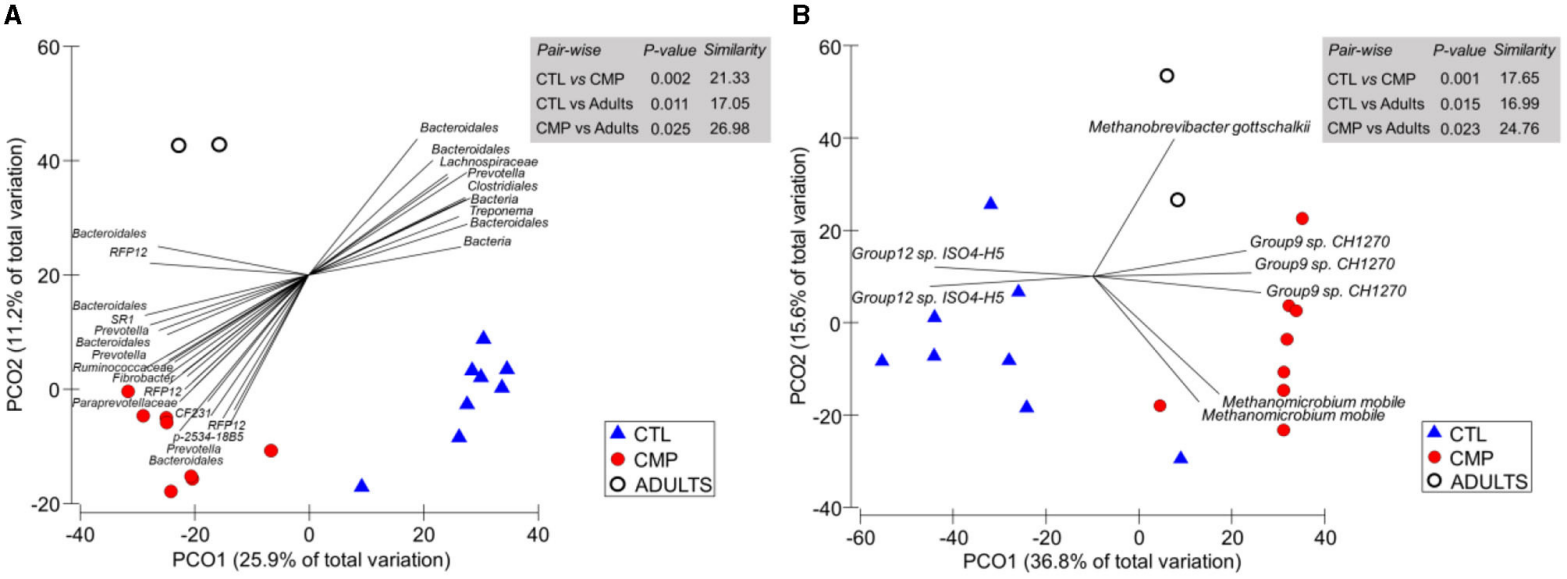
Principal coordinate analysis of the rumen bacterial **(A)** and methanogen **(B)** communities in goat kids (*n* = 8) reared in the absence (CTL) or presence of adult companions (CMP) at 7 weeks of age. Tripod vectors are included to describe the direction and intensity of the most discriminant ASVs (based on Spearman's correlation > 0.8). Samples from adult companions are also shown (Adults). Pair-wise PERMANOVA values are provided in gray boxes based on the Bray–Curtis dissimilarity.

PERMANOVA analysis showed a clear effect of the treatment on the rumen prokaryotic community structure (Figure 2). Pair-wise comparisons identified a substantial difference between the CMP and CTL rumen bacterial (*p* = 0.002) and methanogen communities (*p* = 0.001), indicating a relatively low level of similarity among these treatments (21.33 and 17.65%, respectively). Both CMP and CTL kids differed in the bacterial and methanogen community structure with the adult goats (*p* < 0.05); however, the level of similarity was higher between CMP kids and adult goats (27.0 and 24.8% for bacteria and methanogens) than between CTL kids and adult goats (17.1 and 17.0%, respectively). These differences were also noted in the subsequent PCoA analysis for the bacterial community (Figure 2A), in which the PCO1 axis (which explained 25.9% of the total variation) sorted the samples by the treatment factor. A similar pattern was depicted in the PCoA for the methanogen community, where the most discriminant axis (PCO1 explaining 36.6% of the total variation) separated CTL kids from CMP kids and adults. Several bacterial groups such as *Prevotella, Fibrobacter*, CF231, *Paraprevotellaceae, Ruminococcaceae*, RFP12, and some *Bacteroidales* ASVs positively correlated with the rumen community structure of CMP kids, whereas others such as *Treponema, Clostridiales*, or *Lachnospiraceae* ASVs negatively correlated with the CMP bacterial community (Figure 2A). Within the methanogen community, the most discriminant ASVs were *Methanomicrobium mobile* and Group 9 sp for the CMP kids, Group 12 sp for the CTL kids, and *Methanobrevigacter gottschalkii* for the adult goats (Figure 2B).

The assessment of the relative abundance of the rumen prokaryotic taxa showed that 7 out of the 20 most abundant bacterial families (Table 3; Supplementary Table 3) and 5 out of 10 methanogens species (Table 4) denoted differences between CMP and CTL kids. *Prevotella* was by far the most abundant genus in the rumen of goat kids regardless of treatment (37% of the total sequences). At phylum level, *Bacteroidetes* tended to be more abundant in CMP kids in contrast with *Firmicutes*, whose abundance was higher in CTL kids. CTL kids also harbored a higher presence of *Proteobacteria* and *Spirochaetes* (10.8% between the two phyla in CTL kids, in comparison with 4.32% in CMP kids). Other than comparisons between phyla, CMP kids had higher abundance of several rumen bacterial taxa including *Bacteroidaceae, Veillonellaceae, Erysipelotrichaceae, F16, RFP12, Selenomonas, Succinispira, Bulleidia, Spirochaeta*, and *Dethiosulfovibrio*. On the contrary, *Rhodocyclaceae, Desulfovibrionaceae, Spirochaetaceae*, R4_41B family, *Georgfuchsia*, and *Treponema* showed a greater abundance in CTL kids. With regard to methanogens, the species *Group9_ISO4_G1, M. mobile*, and *Group9_sp_CH1270* were more abundant in CMP kids, while *Group12_ISO4_H5* was more prevalent in CTL kids. Most of the taxa which were significantly more abundant in CMP than in CTL kids were also numerically more abundant in the rumen of the adult companions than in the CTL kids.

**Table 4:** Abundance of rumen methanogens in the rumen of goat kids reared in the absence (CTL) or presence of adult companions (CMP) at 7 weeks of age (*n* = 8).

	**Treatments**				
	**CTL**	**CMP**	**Adults^a^**	**SEM**	***p*** **-value^b^**
*Methanobrevibacter gottschalkii*	12.2	9.60	44.9	1.91	0.442
*Group12_ISO4_H5*	48.2	0.76	0.00	7.370	<0.001
*Group8_WGK1*	2.48	0.00	2.04	0.703	0.234
*Group9_ISO4_G1*	11.4	37.1	13.3	4.650	0.002
*Group10_sp*	1.61	1.51	6.12	0.489	0.878
*Group9_sp_CH1270*	0.89	18.4	1.53	3.080	0.002
*Methanomicrobium mobile*	5.48	26.5	0.00	5.040	0.007
*Group11_sp_ISO4_G11*	9.26	3.72	14.3	3.180	0.878
Unidentified	8.54	2.21	6.12	1.350	0.007

a *Description of the rumen bacterial community in the adult companions*.

b *p-values for the differences between CTL and CMP kids*.

Spearman correlations were performed to assess the potential implications of changes in rumen meta-taxonomic data on animal physiology and rumen fermentation parameters (Supplementary Table 5). Bacterial, methanogen, and anaerobic fungal abundance, as well as various microbial taxa such as *Fibrobacteres, SR1, TM7 F16*, and *Methanomicrobium* and various protozoa, positively correlated with physiological indicators such as total rumen VFA, blood BHB, and BHB/glucose ratio. The abundance of the phyla SR1, TM7, bacterial diversity, and anaerobic fungi concentration also had a positive correlation with the BHB/Glucose ratio, whereas this ratio negatively correlated with the taxa Rhodocyclaceae, Spirochaetaceae, and Methanobrevibacter. The rumen concentration of protozoa, anaerobic fungi, methanogens, and *Fibrobacteres* positively correlated with BW. Rumen protozoal concentration and abundances of most protozoal groups had a positive correlation with the concentrations of rumen butyrate and blood urea and proteins.

## Discussion

Previous studies with calves have demonstrated that decreasing the milk allowance during the artificial milk feeding period or optimizing the type of solid feed (e.g., presentation form and taste) and feeder location prior to weaning can encourage young ruminants to increase the solid feed intake (1, 4). However, artificial milk feeding in conjunction with early weaning strategies can limit the rumen microbiological colonization by a microbial consortium able to perform the major fermentative and metabolic functions required at weaning and beyond, which may have negative effects on the weaning process (3). Previous works have demonstrated that lambs (35) and goat kids (36) reared with their dams experienced an earlier rumen microbial and physiological development than those artificially reared in the absence of adults. Moreover, we have confirmed that this microbial transfer can be mimicked by inoculating young ruminants with rumen fluid from adult animals (9, 10), promoting an acceleration of the rumen microbiological colonization and favoring the solid feed intake prior to weaning. The present study builds upon these previous findings and investigates an alternative strategy based on the presence of a low number of adult non-lactating companions to be practical under farm conditions, but potentially maintaining similar positive effects.

### Rumen Fermentation

Although individual DMI was not measured in our study, CMP kids showed a higher rumen pH than CTL kids which could be due to a greater forage intake as a result of the presence of rumen protozoa (9, 37). However, there is still controversy on whether the higher solid feed intake could rely on a direct effect derived from a social feeding learning from the adult goats (20) or on an indirect effect driven by the microbial transfer which allows a better digestion of fibrous feeds (38). Even though no major differences were found in the total VFA and ammonia concentrations between CTL and CMP goat kids, a notably higher production of rumen butyrate (+45%) was observed in CMP kids across sampling times. Butyrate has been described as a key fermentation product involved in the rumen and intestinal epithelial development (39, 40) and in the overall health status of young ruminant (41). Butyrate production is a result of starch and cellulose degradation, a process which is greatly associated with the fibrolytic protozoal activity (42, 43), which explains its positive correlation with rumen protozoal abundance in this study. A recent study also reported a similar increase (+52%) in the butyrate molar proportion in naturally reared in comparison with artificially reared lambs (35). Therefore, the increasingly greater butyrate concentration in CMP than in CTL kids over time (from +25% at week 5 to +83% at week 9) could rely on a greater solid feed intake which could favor a smooth transition from liquid to solid feeding as previously reported (9). Similarly, the greater levels of branched-chain VFA (+20.4%) and twice higher ammonia concentration noted in CMP than in CTL kids across sampling times suggest a higher rumen proteolysis (44). As already reported in previous works where either young (9) or adult (45) ruminants were inoculated with rumen fluid, the higher concentration of ammonia and branched-chain VFA was associated with a higher breakdown of dietary and bacterial protein by rumen protozoa (46). The higher plasma BHB concentration (+41%) noted in CMP than in CTL kids across all sampling times can be justified by a greater ruminal butyrate concentration. This increase in BHB concentration has been associated with greater fermentative activity, VFA absorption, and bio-transformation in the rumen wall, as it was also reported when direct rumen microbiota inoculation was performed on young ruminants (9, 15). On the contrary, blood glucose levels in young ruminants decrease with age and can be considered as an indicator of insufficient GIT physiological development (9). In our study, the lower blood glucose concentrations observed in CMP compared with CTL kids at 5 (−10%) and 7 weeks of age (−23%) ascertain that the transition from proto-rumen to matured rumen could have been accelerated in CMP kids (47), possibly as a result of a more developed rumen microbiota and/or feeding behavior.

### Rumen Eukaryotes

This study showed that the presence of adult goats as companions of CMP kids, in contrast to CTL kids, accelerated the rumen microbial development, leading to the presence of a more complex microbial community characterized by an abundant and diverse protozoal community. Unlike rumen prokaryotes, rumen protozoa are strict anaerobes considered late rumen colonizers which can only be transferred by direct contact with adult animals (48, 49), with the common drinking water area being one of the main sources (6). As a result, CTL kids lacked rumen protozoa throughout the whole experiment. On the contrary, CMP kids harbored a relevant protozoal community at 5 weeks of age, which became more complex over time and therefore more closely resembling the protozoal community observed in the adult companions. The predatory activity on rumen bacteria by the protozoa could explain the similar total bacteria abundance between CTL and CMP kids (46). Despite the protozoal abundance not changing between sampling times, *Entodiniinae* was by far the predominant rumen protozoa in CMP kids at 5 weeks of age followed by *Diplodiniinae*. However, *Entodiniinae* abundance decreased over time in favor of *Ophryoscolex* and holotrichs (*Isotricha* and *Dasytricha*) resulting in a more diverse protozoal community. The concentration of rumen protozoa and the abundance of some protozoal groups positively correlated with the BW and blood BHB, indicating a positive effect on the rumen function which could favor the fiber degradation in CMP kids after weaning (37). In relation to the rumen anaerobic fungal population, previous studies have shown that young ruminants natural reared on the dam (35) or inoculated with rumen fresh rumen fluid from adult animals (10) had higher rumen fungal diversity (or concentration) and a different community structure than those artificially reared on milk replacer. In our study, the rumen anaerobic fungal concentration positively correlated with rumen acetate and certain indicators of physiological development (i.e., BW and blood BHB); however, no differences between treatments were noted between treatments, possibly because fungi can develop resistant spores that facilitate their transmission without the need of physical contact across animals (50).

### Rumen Prokaryotes

The rumen colonization by prokaryotes has been described as a sequential process that occurs earlier than for eukaryotes (51). The rumen is primarily colonized by facultative anaerobes (mostly *Proteobacteria*) (52, 53), but shortly after the first days of life a great microbial shift takes place as strict anaerobes (i.e., *Firmicutes* and *Bacteroidetes*) get established, making the bacterial community more diverse. This process is particularly evident when animals start ingesting solid diet, and cellulolytic bacteria such as Fibrobacteres and Firmicutes start occupying relevant niches (54). Similarly, rumen methanogens (e.g., *Metanobacteriales* and *Methanomicrobiales*) have also been reported to colonize the rumen from the first week of age with substantial taxonomic changes thereafter (55, 56). In our study, a higher rumen bacterial diversity (+132 ASVs) was observed in CMP kids at 7 weeks of age compared to CTL kids. Similar increments in the bacterial diversity have been found in naturally reared lambs compared with artificially reared lambs (35, 36) and in young ruminants inoculated with rumen fluid (10, 57).

Although the primers used in our study have been validated to simultaneously study the rumen bacterial and methanogen communities (29, 58), the lower coverage observed for the latter community may limit the identification of low-abundance methanogens. A small but consistent number of methanogen sequences were detected in our study, representing ~0.6% of the total sequences and being in agreement with the proportion expected in the rumen (59). Despite this methodological limitation, our study showed that the CMP kids tended to have higher methanogen diversity (Evenness and Simpson index) than the CTL kids. The presence of rumen protozoa has been associated with greater bacterial and methanogen diversities due to the presence of an important epi- and endo-symbiotic prokaryotic community associated with rumen protozoa (37).

The study of the core rumen community has been proposed as a useful approach to describe the colonization pattern (52, 60). Our study showed that a greater proportion of the bacterial and methanogen core communities were shared between the adult companion goats and CMP kids than with CTL kids. Moreover, a great number of bacterial ASVs remained present only in the adult animals, indicating that the rumen prokaryotic colonization process is still far from being completed at weaning. This observation is in line with previous observations (52), which suggested that the rumen colonization is a long-lasting and continuous process which takes several years to be fully accomplished. Similarly, a substantial number of prokaryotic ASVs were only present in either CMP or CTL kids, indicating that the shared environment can determine and homogenize the rumen microbial composition as a result of the cross-contamination between animals. The greater treatment-specific core community observed in CMP kids was most likely a result of a microbiological enriched environment due to the presence of several microbial sources from the adult goats such as feces, udder or skin (5).

Regarding the community structure of the rumen microbiota, the higher level of similarity observed between the adult animals and CMP kids, in comparison to CTL kids, reinforces the existence of a microbial transmission. Moreover, some of the most discriminant ASVs between CMP and CTL kids (e.g., *Prevotella, Ruminococcaceae*, and *Fibrobacter*) have been identified as part of the adult core rumen microbiome (61) and/or indicators of the rumen microbial development (10). This study also noted differences in the relative abundance of some microbes, which may indicate the presence of a more mature rumen microbiota in CMP than in CTL kids (10, 17). Most of the bacterial taxa with higher abundance in CTL kids belonged to *Proteobacteria* and *Spirochaetes* (mainly sugar-degrading or sulfate-reducing bacteria), indicating that an important number of early colonizers were still present in the rumen of CTL kids at 7 weeks of age. On the contrary, CMP kids had higher abundances of *Bacteroidetes* (+11%) which has been reported as an indicator of a rumen microbiota suited to digest grain diets (59) as well as being regarded as a phylum with a high lignocellulolytic and hemicellulolytic activity (62). Furthermore, other relevant taxa such as *Tenericutes, Cyanobacteria*, and *Veillonellaceae* were also increased in CMP kids and have been recently correlated with indicators of rumen functional development such as forage and solid intake, presence of protozoa, and higher bacterial, protozoal, and methanogen diversity (10). In this sense, the greater presence of late colonizer *Selenomonas* in CMP kids (52) could contribute to enhance fiber digestion when partnered with *F. succinogenes* (63). The present study did not find a clear transmission pattern of individual methanogen species from adult companions to CMP goat kids. However, the increased levels of *M. mobile* and *Group9* in CMP kid are consistent with the changes found in goat kids inoculated with fresh rumen fluid, being both methanogen species positively correlated with bacterial diversity and rumen development indicators (10). This observation, along with the greater total methanogen abundance (which positively correlated with BW and BHB) and a tendency to greater diversity in this group, indicates that CMP kids may host a more mature rumen methanogen community at weaning, possibly shaped by the presence of a complex protozoal community as previously described (64–66).

These observations demonstrated that rearing young ruminants in the presence of adult goats accelerated the rumen microbial and functional development with potential benefits in the transition from the liquid to solid feed. A previous study with artificially reared kids pointed out a milk replacer cost of 24€ per kid when animals are weaned at 7 weeks of age, but with the possibility of decreasing this cost by 20 or 35% if kids are weaned at 5 or 6 weeks of age, respectively (9). The feeding practices used in this study and based on *ad libitum* milk feeding and late weaning did not help to visualize the full potential of this strategy in terms of animal performance, as noted in similar studies (9, 20). However, the implementation of more challenging farm conditions such as low milk allowance or early weaning practices (1) could potentially enhance the positive effects derived from a greater rumen microbial and physiological development.

## Conclusions

Overall, these findings revealed that the presence of adult goats as companions facilitated the rumen microbial transfer of protozoa and specific methanogens and bacterial taxa to young ruminants accelerating the rumen microbial and functional development. This strategy could facilitate the transition from liquid to solid feed with potential positive effects on the weaning process. Further studies are needed to investigate the short- and long-term effects of young ruminants reared in the presence of non-lactating adults on the rumen microbiota and animal performance under more challenging farm conditions.

## Data Availability Statement

The datasets presented in this study can be found in online repositories. The names of the repository/repositories and accession number(s) can be found in the article/Supplementary Material.

## Ethics Statement

The animal study was reviewed and approved by Ethical Committee for Animal Research (EEZ-CSIC).

## Author Contributions

DY-R, AB, and AM-G: conceptualization and funding acquisition. JP-H, AB, and EJ: methodology and data acquisition. JP-H and AB: data curation and software. JP-H: writing original draft. AB and DY-R: writing, review, editing, and project administration. All authors have read and approved the final manuscript.

## Funding

This work was supported by the European Union's Horizon 2020 Research and Innovation program under grant agreement no. 818368 (MASTER) and by the Spanish Government through the project AGL2017-86938-R. JP-H has a grant from the Training Program for Academics, Madrid, Spain (FPU16/01981) and AB is a Ramón y Cajal Fellow [RYC2019-027764-I/AEI/10.13039/501100011033] from the Spanish Research Agency.

## Conflict of Interest

The authors declare that the research was conducted in the absence of any commercial or financial relationships that could be construed as a potential conflict of interest.

## Publisher's Note

All claims expressed in this article are solely those of the authors and do not necessarily represent those of their affiliated organizations, or those of the publisher, the editors and the reviewers. Any product that may be evaluated in this article, or claim that may be made by its manufacturer, is not guaranteed or endorsed by the publisher.
