# Supplementary material for: Presence of Adult Companion Goats Favors the Rumen Microbial and Functional Development in Artificially Reared Kids
Source: Front Vet Sci. 2021 Sep 7;8:706592. doi: 10.3389/fvets.2021.706592 (PMC8453066; doi:10.3389/fvets.2021.706592)
Supplement: Supplementary file 1 [file Data_Sheet_1.docx]

**SUPPLEMENTARY MATERIAL**

**Presence of adult companion goats favors the rumen microbial and functional development in artificially reared kids**

Palma-Hidalgo J.M., Yáñez-Ruiz D.R., Jiménez E., Martín-García A.I. and Belanche A.

**Supplementary Table S1.** Chemical composition (in % DM) of the feeds.

|  | Milk replacer^1^ | Starter concentrate^2^ | Oats  hay |
| --- | --- | --- | --- |
| Organic matter | 92.8 | 93.1 | 90.2 |
| Crude protein | 24.0 | 12.9 | 8.1 |
| Ether extract | 22.0 | 3.2 | 2.2 |
| Neutral-detergent fiber |  | 22.9 | 50.9 |
| Acid-detergent fiber |  | 9.12 | 32.6 |

^1^ Declared composition

^2^ Ingredient list: wheat bran, barley, corn, wheat, sunflower seed flour, soybean flour, CaCO_3_, NaCl, vitamin mineral premix and NaCO_3_.

**Supplementary Table S2.** Description of the of the rumen protozoal community in the two adult companions (*n*=8).

|  | Adults^1^ |
| --- | --- |
| Protozoal counts, log10 cells/mL | 5.81 |
| Subf. *Entodiniinae*, % | 75.2 |
| Subf. *Diplodiniinae*, % | 5.06 |
| *Ophryoscolex* spp., % | 10.6 |
| *Isotricha* spp., % | 2.33 |
| *Dasytricha* spp., % | 6.80 |

^1^Average between the two adult companions.

**Supplementary Table S3.** Diversity indexes and abundances of rumen bacterial taxa in the rumen of goat kids reared in absence (CTL) or presence of adult companions (CMP) at 7 weeks of age (n =8).

|  | Treatments | |  |  |  |
| --- | --- | --- | --- | --- | --- |
|  | CTL | CMP | Adults^1^ | SEM | *P*-value^2^ |
| Richness | 216 | 348 | 446 | 20.3 | <0.001 |
| Shannon | 3.910 | 4.670 | 5.290 | 0.135 | <0.001 |
| Evenness | 0.728 | 0.798 | 0.871 | 0.015 | 0.016 |
| Simpson | 0.949 | 0.973 | 0.999 | 0.006 | 0.051 |
| Good’s coverage | 0.991 | 0.994 | 0.999 | 0.002 | 0.502 |
| **Abundance** (%) |  |  |  |  |  |
| *p_Actinobacteria, f_Coriobacteriaceae, g_Olsenella* | 0.124 | 0.036 | 0.823 | 0.025 | 0.079 |
| *p_Bacteroidetes* | 64.20 | 71.50 | 62.60 | 1.960 | 0.093 |
| *f_Barnesiellaceae, g_Barnesiella* | 3.960 | 0.579 | 0.639 | 1.040 | 0.115 |
| *f_Paraprevotellaceae* | 3.230 | 5.770 | 2.400 | 0.704 | 0.059 |
| *g_CF231* | 0.346 | 0.621 | 1.290 | 0.090 | 0.195 |
| *g_YRC22* | 0.136 | 0.029 | 0.064 | 0.058 | 0.643 |
| *f_Bacteroidaceae, g_BF311* | 0.179 | 0.615 | 1.57 | 0.108 | 0.027 |
| *f_Porphyromonadaceae* | 0.117 | 0.016 | 0.113 | 0.031 | 0.130 |
| *f_Prevotellaceae, g_Prevotella* | 35.30 | 38.70 | 19.90 | 2.370 | 0.753 |
| *f_RF16* | 0.034 | 0.282 | 0.049 | 0.107 | 0.125 |
| *f_Sphingobacteriaceae, g_Pedobacter* | 0.349 | 0.154 | 0.049 | 0.119 | 0.746 |
| *p_Chloroflexi* | 0.010 | 0.021 | 0.042 | 0.120 | 0.334 |
| *p_Cyanobacteria* | 0.015 | 0.081 | 0.054 | 0.164 | 0.021 |
| *p_Elusimicrobia* | 0.162 | 0.393 | 0.108 | 0.078 | 0.290 |
| *f_Elusimicrobiaceae* | 0.154 | 0.205 | 0.049 | 0.054 | 0.742 |
| *p_Fibrobacteres, g_Fibrobacter* | 2.180 | 2.230 | 8.590 | 0.451 | 0.916 |
| *p_Firmicutes* | 16.60 | 13.70 | 21.40 | 0.837 | 0.059 |
| *f_Clostridiaceae* | 0.167 | 0.121 | 0.196 | 0.037 | 0.916 |
| *g_Clostridium* | 0.383 | 0.245 | 0.771 | 0.052 | 0.248 |
| *f_Lachnospiraceae* | 3.240 | 1.640 | 4.340 | 0.537 | 0.345 |
| *g_Lactonifactor* | 1.160 | 0.232 | 0.277 | 0.403 | 0.318 |
| *f_Ruminococcaceae* | 7.310 | 5.090 | 5.710 | 0.705 | 0.093 |
| *g_Ruminococcus* | 0.390 | 0.432 | 0.766 | 0.073 | 0.753 |
| *g_Papillibacter* | 0.299 | 0.057 | 0.044 | 0.0667 | 0.125 |
| *g_Sporobacter* | 0.301 | 0.086 | 0.213 | 0.075 | 0.267 |
| *f_Veillonellaceae* | 0.677 | 1.398 | 1.210 | 0.184 | 0.027 |
| *g_Selenomonas* | 0.139 | 0.325 | 0.147 | 0.068 | 0.035 |
| *g_Succinispira* | 0.009 | 0.685 | 0.850 | 0.142 | 0.001 |
| *f_Erysipelotrichaceae* | 1.030 | 1.470 | 0.747 | 0.269 | 0.046 |
| *g_Bulleidia* | 0 | 0.121 | 0.054 | 0.036 | 0.004 |
| *p_Lentisphaerae* | 0.207 | 0.242 | 0.306 | 0.044 | 0.834 |
| *f_Victivallaceae* | 0.204 | 0.242 | 0.267 | 0.044 | 0.834 |
| *g_Victivallis* | 0.063 | 0.065 | 0.054 | 0.016 | 0.832 |
| *p_Proteobacteria* | 4.240 | 2.230 | 0.857 | 1.040 | 0.338 |
| *f_Neisseriaceae* | 0.052 | 0.045 | 0 | 0.009 | 0.431 |
| *f_Rhodocyclaceae* | 0.214 | 0.051 | 0.024 | 0.048 | 0.059 |
| *g_Georgfuchsia* | 0.209 | 0.046 | 0 | 0.048 | 0.046 |
| *f_Desulfovibrionaceae* | 0.439 | 0.237 | 0.443 | 0.057 | 0.036 |
| *f_Succinivibrionaceae* | 0.581 | 0.480 | 0.323 | 0.196 | 0.529 |
| *g_Succinivibrio* | 0.277 | 0.040 | 0.147 | 0.108 | 0.324 |
| *p_Spirochaetes* | 6.570 | 2.090 | 0.695 | 1.220 | 0.021 |
| *f_Sphaerochaetaceae, g_Sphaerochaeta* | 0.026 | 0.059 | 0 | 0.019 | 0.666 |
| *f_Spirochaetaceae* | 5.050 | 1.250 | 0.409 | 1.020 | 0.021 |
| *g_Spirochaeta* | 0.020 | 0.551 | 0.127 | 0.012 | 0.062 |
| *g_Treponema* | 2.040 | 0.582 | 0.152 | 0.389 | 0.036 |
| *p_SR1* | 0 | 0.114 | 0.260 | 0.038 | 0.001 |
| *p_Synergistetes* | 0.072 | 0.187 | 0.176 | 0.034 | 0.059 |
| *f_Dethiosulfovibrionaceae* | 0.067 | 0.181 | 0.166 | 0.035 | 0.093 |
| *g_Dethiosulfovibrio* | 0.007 | 1.200 | 0.716 | 0.035 | 0.004 |
| *p_Tenericutes* | 0.042 | 0.097 | 0.105 | 0.022 | 0.088 |
| *p_TM7, f_F16* | 0.080 | 0.231 | 0.037 | 0.037 | 0.021 |
| *p_Verrucomicrobia* | 1.280 | 3.350 | 0.764 | 0.620 | 0.074 |
| *f_R4_41B* | 0.683 | 0.395 | 0.071 | 0.145 | 0.207 |
| *f_RFP12* | 0.585 | 2.95 | 0.597 | 0.577 | 0.016 |

^1^Description of the rumen bacterial community in the two adult companions.

^2^*P*-values for the differences between CTL and CMP kids. Only taxa with an average abundance > 0.05% are shown. *p*=phylum; *f*=family; *g*=genus

**Supplementary Table S4.** Diversity indexes of the methanogen community in the rumen of goat kids reared in absence (CTL) or presence of adult companions (CMP) at 7 weeks of age (n=8).

|  | Treatments | |  |  |  |
| --- | --- | --- | --- | --- | --- |
|  | CTL | CMP | Adults^1^ | SEM | *P*-value^2^ |
| Richness | 17.6 | 21.1 | 18.5 | 2.080 | 0.439 |
| Shannon | 2.18 | 2.48 | 2.38 | 0.124 | 0.190 |
| Evenness | 0.77 | 0.83 | 0.82 | 0.021 | 0.064 |
| Simpson | 0.80 | 0.87 | 0.87 | 0.023 | 0.103 |
| Good’s coverage | 0.741 | 0.642 | 24.4 | 0.0292 | 0.237 |

^1^Description of the rumen bacterial community in the adult companions.

^2^*P*-values for the differences between CTL and CMP kids. Only taxa with an average.

**Supplementary Table S5.** Correlations between the rumen microbiota and rumen fermentation and physiological parameters (only Spearman correlations with *ρ* > 0.4 and *P* < 0.01 are shown).

| Correlations | Acetate | Propionate | Isobutyrate | Butyrate | Isovalerate | Valerate | VFA | BW | ADG-pre-weaning | ADG-post-weaning | Blood BHB | Blood glucose | Blood urea | Blood proteins | BHB/Glucose |
| --- | --- | --- | --- | --- | --- | --- | --- | --- | --- | --- | --- | --- | --- | --- | --- |
| Bacterial concentration |  |  |  | 0,45 | -0,40 |  | 0,45 |  |  |  | 0,85 | 0,57 |  | 0,43 | 0,65 |
| Bacterial ASV |  |  | -0,40 |  |  | -0,45 | 0,41 |  |  |  |  | -0,49 | 0,55 |  | 0,42 |
| p__Cyanobacteria |  |  |  |  |  |  |  |  |  |  |  |  | 0,45 |  |  |
| p__Fibrobacteres |  |  |  |  |  |  |  | 0,47 | 0,47 |  | 0,40 |  |  |  |  |
| p__Firmicutes |  |  |  |  |  |  |  |  |  |  |  |  |  | -0,41 |  |
| p__Spirochaetes |  |  |  |  |  |  |  |  | -0,44 |  | -0,40 | 0,50 | -0,67 |  | -0,50 |
| p__SR1 |  |  |  |  |  | -0,40 |  |  |  |  | 0,53 | -0,59 | 0,62 |  | 0,61 |
| p__Synergistetes |  |  | -0,41 |  |  |  |  |  |  |  |  |  |  |  |  |
| p__TM7 |  | 0,43 | -0,44 |  |  |  |  |  |  |  |  | -0,47 |  |  | 0,52 |
| p__Verrucomicrobia |  |  |  |  |  |  |  |  |  |  |  |  | 0,42 |  |  |
| f__[Barnesiellaceae] |  |  |  |  |  |  |  |  |  |  |  |  | -0,42 |  |  |
| f__Ruminococcaceae |  |  |  |  |  |  |  |  |  |  |  |  | -0,58 |  |  |
| f__Erysipelotrichaceae |  |  |  |  |  |  |  |  |  |  |  |  | 0,50 |  |  |
| f__Rhodocyclaceae |  |  |  |  |  |  |  |  |  |  | -0,43 | 0,43 |  |  | -0,50 |
| f__Succinivibrionaceae |  |  |  |  |  |  |  |  |  |  |  |  | 0,40 |  |  |
| f__Spirochaetaceae |  |  |  |  |  |  |  |  | -0,41 |  | -0,43 | 0,56 | -0,67 |  | -0,53 |
| f__Dethiosulfovibrionaceae |  |  |  |  |  |  |  |  |  |  |  |  |  |  |  |
| f__F16 |  | 0,43 | -0,44 |  |  |  |  |  |  |  | 0,53 | -0,47 |  |  | 0,52 |
| f__R4-41B |  | -0,41 |  |  |  |  |  |  |  |  |  |  |  |  |  |
| Methanogens concentration | -0,56 | 0,52 |  | 0,53 |  |  | 0,40 | 0,93 |  |  | 0,85 | 0,48 | 0,69 | 0,69 | 0,63 |
| g__Methanobrevibacter |  |  |  |  |  |  |  |  |  |  | -0,74 | 0,74 | -0,45 | 0,40 | -0,79 |
| g__Group12 |  |  | 0,44 |  |  | 0,46 | -0,45 |  |  |  |  |  | -0,58 |  |  |
| g__Group9 |  |  |  |  |  |  |  |  |  |  |  |  | 0,67 |  |  |
| g__Methanomicrobium |  |  |  |  |  |  |  |  |  | -0,52 | 0,40 |  | 0,50 |  |  |
| Protozoal concentration | -0,60 |  |  | 0,55 |  |  |  | 0,57 |  |  |  |  | 0,78 | 0,99 |  |
| Protozoa (optical count) | -0,55 |  |  | 0,43 |  |  |  | 0,64 |  |  |  |  | 0,57 | 0,81 |  |
| Entodinium | -0,44 |  |  |  |  |  |  | 0,54 |  |  | 0,45 |  | 0,75 | 0,95 |  |
| Diplodiniinae | -0,40 |  |  |  |  |  |  | 0,68 |  |  |  | 0,46 | 0,91 | 0,99 |  |
| Ophryoscolex |  |  |  |  |  |  |  | 0,65 |  |  |  |  |  | 0,51 |  |
| Isotricha |  |  |  |  |  |  |  |  |  |  | 0,42 | 0,59 | 0,71 |  | 0,40 |
| Dasytricha |  |  |  |  |  |  |  | 0,87 |  |  | 0,60 |  |  |  |  |
| Anaerobic fungal concentration | 0,43 |  |  |  |  |  |  | 0,40 |  |  | 0,90 | 0,77 |  |  | 0,85 |
